# 18F-Fluorodeoxyglucose Positron Emission Tomography/Computed Tomography in Large-Vessel Vasculitis During Active and Inactive Disease Stages Is Associated with the Metabolic Profile, but Not the Macrophage-Related Cytokines: A Proof-of-Concept Study

**DOI:** 10.3390/cells13221851

**Published:** 2024-11-08

**Authors:** Dimitris Anastasios Palamidas, Georgios Kalykakis, Dimitra Benaki, Loukas Chatzis, Ourania D. Argyropoulou, Panagiota Palla, Antonia Kollia, Pavlos Kafouris, Marinos Metaxas, Andreas V. Goules, Emmanuel Mikros, Konstantinos Kambas, Constantinos D. Anagnostopoulos, Athanasios G. Tzioufas

**Affiliations:** 1 Department of Pathophysiology and Joint Academic Rheumatology Program, School of Medicine, National and Kapodistrian University of Athens, 11526 Athens, Greece; 2 Department of Informatics, Ionian University, 49100 Kerkyra, Greece; 3 PET-CT Department & Preclinical Imaging Unit, Center for Experimental Surgery, Clinical & Translational Research, Biomedical Research Foundation of the Academy of Athens, 11527 Athens, Greece; 4 Department of Pharmaceutical Chemistry, School of Pharmacy, National and Kapodistrian University of Athens, 15771 Athens, Greece; 5 Research Institute for Systemic Autoimmune Diseases, 11526 Athens, Greece; 6 Laboratory of Immunobiology, Center for Clinical, Experimental Surgery and Translational Research, Biomedical Research Foundation of the Academy of Athens, 11527 Athens, Greece; 7 Athena Research and Innovation Center in Information Communication & Knowledge Technologies, 15125 Marousi, Greece; 8 Laboratory of Molecular Genetics, Department of Immunology, Hellenic Pasteur Institute, 11521 Athens, Greece

**Keywords:** giant cell arteritis, large vessel vasculitis, cytokines, metabolomics, 18F-FDG PET-CT

## Abstract

Giant cell arteritis (GCA) is an autoimmune/autoinflammatory disease affecting large vessels in patients over 50 years old. The disease presents as an acute inflammatory response with two phenotypes, cranial GCA and large-vessel vasculitis (LV)-GCA, involving the thoracic aorta and its branches. 18F-fluorodeoxyglucose positron emission tomography/computed tomography (18F-FDG PET-CT) is among the imaging techniques contributing to diagnosing patients with systemic disease. However, its association with soluble inflammatory markers is still elusive. This proof-of-concept study aims to identify novel soluble serum biomarkers in PET/CT-positive patients with LV-GCA and associate them with active (0 months) and inactive disease (6 months following treatment), in sequential samples. The most-diseased-segment target-to-background ratio (TBR_MDS_) was calculated for 13 LV-GCA patients, while 14 cranial GCA and 14 Polymyalgia Rheumatica patients with negative initial PET/CT scans served as disease controls. Serum macrophage-related cytokines were evaluated by cytometric bead array (CBA). Finally, previously published NMR/metabolomics data acquired from the same blood sampling were analyzed along with PET/CT findings. TBR_MDS_ was significantly increased in active versus inactive disease (3.32 vs. 2.65, *p* = 0.006). The analysis identified nine serum metabolites as more sensitive to change from the active to inactive state. Among them, choline levels were exclusively altered in the LV-GCA group but not in the disease controls. Cytokine levels were not associated with PET/CT activity. Combining CRP, ESR, and TBR_MDS_ with choline levels, a composite index was generated to distinguish active and inactive LV-GCA (20.4 vs. 11.62, *p* = 0.001). These preliminary results could pave the way for more extensive studies integrating serum metabolomic parameters with PET/CT imaging data to extract sensitive composite disease indexes useful for everyday clinical practice.

## 1. Introduction

Large vessel vasculitides (LVVs) constitute a heterogeneous group of autoimmune/autoinflammatory diseases, affecting mainly the aorta, the aortic arch, and its branches. Giant cell arteritis (GCA) is considered the prototype LVV, particularly in older adults, and presents primarily with two different phenotypes: (i) disease limited to temporal arteries (limited form, cranial GCA) and (ii) disease extending beyond, affecting mainly the thoracic aorta and its branches (systemic form, LV-GCA) [1]. The disease is considered a prototypic delayed/type 4 hypersensitivity reaction in both acute and chronic form. Indeed, the inflamed vessels are infiltrated by activated macrophages, Th1, Th17, B cells, and neutrophils that orchestrate a robust inflammatory response insulting the vessel wall [2].

In recent years, several imaging tools have been developed to depict the extent of the disease [3]. Among them, 18F-fluorodeoxyglucose positron emission tomography/computed tomography (18 F-FDG PET/CT, hereafter called PET/CT) is considered one of the most used tools both for diagnosis and assessment of disease severity. PET/CT not only locates inflammatory isles within the vasculature bed, but also provides quantitative information on the metabolic activity of immune cells (i.e., activated macrophages, giant cells, and T-cells) [4], distinguishing vasculitic from atherosclerotic lesions. Several studies have assessed the relationship between PET/CT findings with disease activity and remission. Recently, the 2022 American College of Rheumatology/EULAR GCA classification criteria emphasized the use of PET/CT as well as other imaging modalities, such as vascular ultrasound and MRI, for use in clinical practice [5]. However, the standardization of scoring methods in PET/CT remains an unmet need in the field [6]. In addition, the relationship between PET/CT-related markers and soluble factors of inflammatory response has not yet been investigated in the context of LV-GCA.

In this study, we sought to investigate the relationship between disease activity assessed by PET/CT and soluble macrophage-related inflammatory markers, metabolites, and lipids in the serum of LV-GCA patients at the active and inactive disease states. Such an attempt is anticipated to describe better this set of patients during each disease state and create new stratification tools, based on the combination of serum biomarker and vascular imaging data.

## 2. Materials and Methods

### 2.1. Study Cohort

For the current study, 41 newly diagnosed patients with GCA and PMR who were followed up at the rheumatology outpatient clinic of the Department of Pathophysiology in Laiko General Hospital were prospectively recruited between 2021 and 2023. All patients had to fulfill the 2012 American College of Rheumatology (ACR)/European Alliance of Associations for Rheumatology (EULAR) classification criteria for PMR [7] or the 1990 American College of Rheumatology/EULAR classification criteria for GCA [8]. GCA patients with negative vascular findings on 18-FDG-PET/CT scans at diagnosis were classified as having cranial GCA (*n* = 14) while those with positive vascular findings on 18-FDG-PET/CT as LV-GCA (*n* = 13). For patients with PMR diagnosis, the exclusion of underlying vasculitis was based on a negative temporal artery biopsy and negative 18-FDG-PET/CT scan findings in the arterial tree (6/14 had positive PET/CT findings consistent with PMR diagnosis). Using an identical methodology, the absence of LV involvement in patients with cranial GCA was confirmed by 18-FDG-PET/CT imaging. Each LV-GCA patient underwent 2 sequential PET/CT scans: one at the time of diagnosis (active phase) and the second 6 months after treatment initiation when patients were in remission (inactive phase). The 14 cranial GCA and the 14 PMR patients served as controls and underwent PET/CT scans only at the time of diagnosis. Blood samples were also collected from all participants at diagnosis before treatment initiation (active phase) and 6 months after glucocorticoid (GC) treatment (inactive phase) to measure acute phase reactants and inflammatory cytokines.

Active disease for GCA and PMR patients was defined as the presence of both clinical symptoms and increased acute phase reactants (ESR > 20 mm/h and CRP > 5 mg/L). The opposite definition was applied to define disease remission. All participants were in complete remission after 6 months of treatment. The study was approved by the Ethics Committee of the School of Medicine, National and Kapodistrian University of Athens, Greece. All participants gave written informed consent before enrollment.

### 2.2. 18F-FDG PET/CT Imaging Protocol

Following overnight fasting for ≥6 h and if blood glucose levels were <180 mg/dL before radiotracer injection, participants underwent PET/CT imaging. Ninety minutes following intravenous administration of 18F-FDG (3.7 MBq per kg patient weight), PET/CT (Biograph, Siemens, Forchheim, Germany) imaging from the top of the skull to the mid-thigh level was performed with the patient placed in supine position. Attenuation correction and co-registration were performed with a low dose non-gated non-contrast enhanced CT (120 kV—30–70 mAs) scan. PET images were reconstructed using a standard Iterative Ordered-Subset Expectation Maximization algorithm. The reconstruction scheme consisted of 4 Iterations and 5 Subsets of 440 × 440 image matrix.

### 2.3. 18F-FDG PET/CT Measurements

18F-FDG PET/CT anonymized images were assessed by an experienced reader who was unaware of patients’ diagnoses and laboratory parameters. The degree of arterial uptake was assessed visually relative to liver uptake in the aorta and its branch arteries (subclavian, axillary, carotid, iliac, and femoral arteries) as follows: 0 = no uptake; 1 = less than liver; 2 = same as liver; 3 = greater than liver [9]. Patients with a relative degree of 0 or 1 in all vessels were classified as having a negative PET/CT scan, while those with 2 or 3 had a positive one. PET vascular activity score (PETVAS) based on the aforementioned grading scale was given to each patient and, in addition, semiquantitative image analysis was performed, the basic principles of which have been previously described [6,10]. In brief, cross sections of circular or ellipsoid volumes of interest (VOI) around the arterial wall were manually drawn along the whole vessel in consecutive axial images at intervals of 5 mm for the thoracic and abdominal aorta. For 18F-FDG uptake quantification, the maximum standardized uptake value (SUV_MAX_) based on body weight was recorded as the highest pixel activity within each VOI. To normalize arterial PET signal for blood activity, at least 6 ROIs (3 to 4 mm in diameter) were placed over the superior vena cava (SVC), and the average SUV mean value was calculated. The arterial target-to-background ratio (TBR) was then derived by dividing the mean arterial SUV_MAX_ by the average value of venous SUV_MEAN_ estimated from the SVC. TBR of the abdominal aorta (TBR_ABD_) was calculated as the average value of the suprarenal and infrarenal abdominal aorta. Furthermore, global TBRaorta (TBR_GLOBAL_) was derived by calculating the sum of TBRs of ascending and descending aorta, aortic arch, and suprarenal and infrarenal abdominal aorta divided by 5. TBR of the most diseased aortic segment (TBR_MDS_) was also obtained by selecting the slice with the highest SUV_MAX_ and calculating the mean of the SUV_MAX_ from this and the two neighboring slices. These semiquantitative measurements were subsequently used for generation of composite indices, which are described in detail below.

### 2.4. Cytometric Bead Array

Cytokines were measured using microbead arrays. A pre-validated array of 13 macrophage-related biomarkers was used from BioLegend (San Diego, CA, USA; Cat# 740502). The biomarkers tested were as follows: IL-12p70, TNF-α, IL-6, IL-4, IL-10, IL-1β, Arginase, CCL17(TARC), IL-1RA, IL-12p40, IL-23, IFN-γ, CXCL10 (IP-10). Samples were processed in duplicate and analyzed per the manufacturer’s instructions. Beads were assayed using a FACSCalibur™ (Becton Dickinson, Franklin Lakes, NJ, USA); analysis of all fcs files to determine protein concentrations was performed using BioLegend Qognit cloud-based software (Biolegend, San Diego, CA, USA).

### 2.5. Metabolomics Data

In the current study, a part of the serum NMR metabolomics dataset from the same GCA and PMR patients, already published recently by our team, was utilized for further analysis [11]. More precisely, NMR metabolomics data were available for the 12 LV-GCA patients, 14 cranial GCA patients, and 14 PMR patients included in the current study. The corresponding NMR data were used to investigate plausible correlations between the features of the two different techniques. The NMR data of the specific individuals include 21 metabolites (identified and quantified in the SMolESY platform using 1D-NOESY NMR spectra) and 20 lipids (derived from LED spectra) and are presented in Supplementary Tables S1 and S2.

### 2.6. Composite Index Generation

Three composite index metrics were formed combining PET/CT measurements, CRP levels, ESR levels, and metabolite levels that demonstrated significant differences between activity and inactivity. All of the metrics, CRP, ESR, and choline levels were transformed into categorical values (Supplementary Table S3) to quantify in the same scale. Composite index 1 is the sum of TBR_MDS_, CRP—categorical, and ESR—categorical. Composite index 2 is the sum of TBR_MDS_, CRP—categorical, ESR—categorical, and choline—categorical. Finally, composite index 3 is the sum of CRP—categorical, ESR—categorical, and choline—categorical.

### 2.7. Statistical Analysis

Quantitative data are presented as medians and interquartile range (IQR), whereas qualitative variables as absolute and relative frequencies. For comparing non-parametric continuous variables and after implementing the Shapiro–Wilk normality test, the Wilcoxon signed-rank test was used for statistical analysis. A two-tailed *p* < 0.05 was considered to indicate statistical significance. Spearman’s correlation coefficient was used to assess correlations. For comparisons among multiple groups, a non-parametric Kruskal–Wallis test was used and Dunn’s test correction method was employed. Analysis was conducted using GraphPad Prism software for Mac (version 10.1.1) (GraphPad Software, San Diego, CA, USA). PCA analysis was performed using Python (version 3.8.19) and the scikit-learn package (version 1.4.2).

## 3. Results

### 3.1. PET/CT Imaging Can Discriminate Between Disease Activity and Remission in LV-GCA

**Figure 1:**
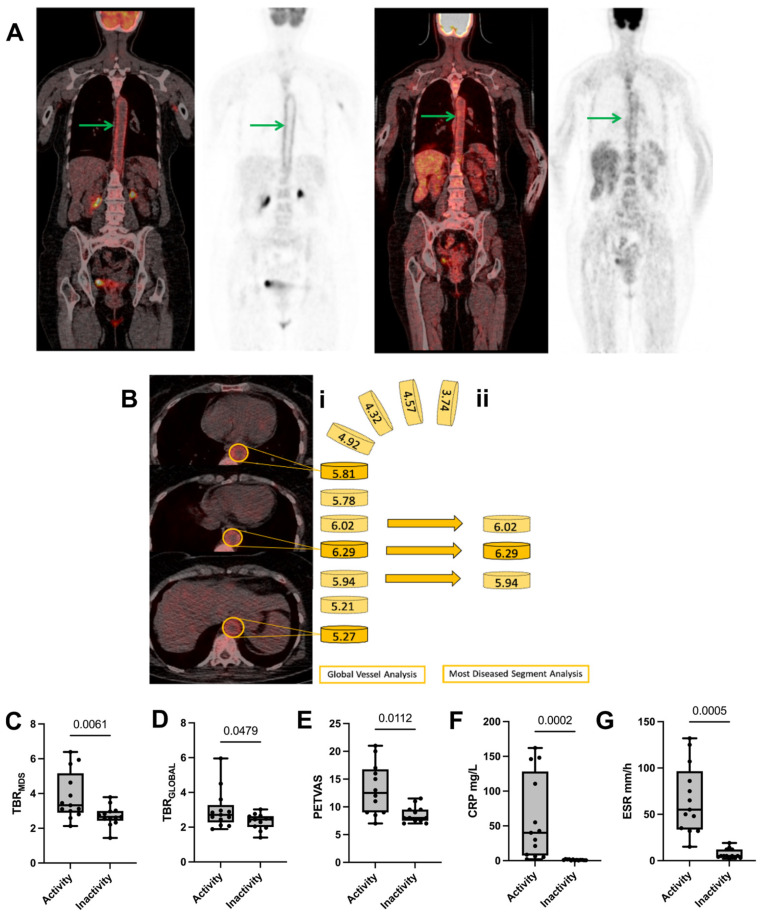
Disease activity and inactivity markers studied in LV-GCA patients. (**A**) A representative imaging result of one female patient who underwent FDG-PET/CT showed increased FDG uptake in the descending thoracic aorta (green arrows) at the first scan (**left panel**) (average TBR value of 6.08), compared to the uptake in the second scan (**right panel**) (average TBR value of 2.39). (**B**) TBR_MDS_ calculation—**i.** global aorta and **ii.** most-diseased-segment (MDS) analysis. The global aortic and MDS scores are calculated by summing and averaging each arterial segment’s max FDG-TBR uptake pattern. The TBR_MDS_ score is calculated as a sum of 3 sequential slice values. The comparison of (**C**) TBR_MDS_ values, (**D**) global TBR values, (**E**) PETVAS values, (**F**) C-reactive protein (CRP) in mg/L, and (**G**) Erythrocyte Sedimentation Rate (ESR) in mm/h, between active and inactive disease states for 13 LV-GCA patients is shown above. The first and third quartiles are shown in the lower and upper horizontal lines. The horizontal line in the boxes represents the median value. The exact *p* values are shown in each boxplot.

To address the study’s objectives, patients from three groups were included (Table 1). The main objective of the study was to assess the ability of 18F-FDG-PET/CT to distinguish between the active and inactive phases in patients with LV-GCA. For this purpose, PET/CT imaging data collected at the two pre-defined time points were analyzed and compared accordingly. Patients in the active phase presented higher TBR scores in the aortic arch (TBR_ARC_), abdominal (TBR_ABD_), descending (TBR_DSC_), and global aortic (TBR_GLOBAL_) measurements compared to remission (*p* < 0.05 for all comparisons, Supplementary Table S4). A representation of the TBR_MDS_ measurement is shown in Figure 1A,B.

TBR_MDS_ and TBR_GLOBAL_ were significantly decreased from the active to inactive phase (3.32 vs. 2.65, *p* = 0.006 (Figure 1C) and 2.71 vs. 2.44, *p* = 0.048 (Figure 1D), respectively). PETVAS was also decreased from the active to inactive phase (12.5 vs. 8, *p* = 0.011) (Figure 1E).

The traditional inflammatory markers CRP and ESR confirmed clinical observations, showing a statistically significant reduction from disease activity to the remission state (Figure 1F,G).

### 3.2. Serum Levels of Certain Metabolites and Lipids, but Not Cytokine Levels, Are Altered Significantly in LV-GCA Patients from the Active to Inactive Disease States

To further identify other markers of systemic inflammation related to the active phase of LV-GCA compared to remission, we analyzed and compared serum levels of cytokines, metabolites, and lipids in LV-GCA patients between the two phases.

Interestingly, there were no statistically significant changes in serum levels of macrophage-related cytokines between disease activity and remission among the LV-GCA patients (Supplementary Table S5).

**Figure 2:**
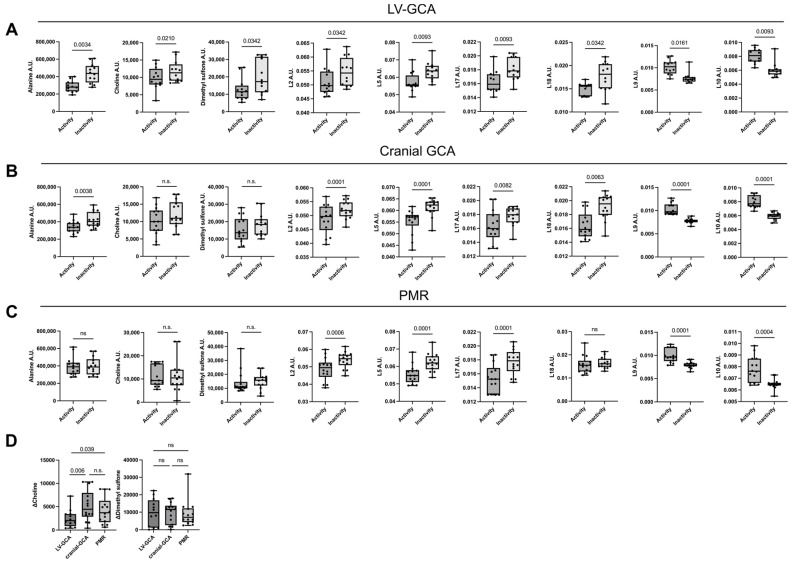
Significantly altered levels of serum metabolites and lipids during active and inactive disease states in 9 LV-GCA patients. (**A**) Boxplots of the 9 significantly altered metabolites and lipids in the LV-GCA patients. (**B**) Only 7 out of 9 metabolites and lipids are significantly altered in the cranial GCA patients. (**C**) Only 6 out of 9 metabolites and lipids are significantly altered in the PMR patients. (**D**) Comparison of Δcholine and ΔDimethyl sulfone between the 3 disease groups. The first and third quartiles are shown in the lower and upper horizontal lines, respectively. The horizontal line in the boxes represents the median value. The exact *p* values are shown in each boxplot. L2, lipids CH3- (mainly HDL); L5, lipids CH2-; L9, N-acetyl glycoproteins (NCH3-) GlycA; L10, N-acetyl glycoproteins (NCH3-) GlycB; L17, cholesterol, methine group CH=CH; L18, methine group CH=CH; cholesterol, unsaturated fatty acids; n.s., non-significant.

Patients demonstrated an increase in serum levels of alanine (*p* = 0.0034), choline (*p* = 0.021), dimethyl sulfone (*p* = 0.0342), and certain lipids [L2: CH3-lipids (*p* = 0.0342), L5: CH2—lipids (*p* = 0.009), unsaturated lipids L17: CH=CH (*p* = 0.0093) and L18: CH=CH (*p* = 0.0342)] as well as a decrease in GlycA (*p* = 0.0161) and GlycB levels (*p* = 0.009) from the active to inactive phase (Figure 2A). The analysis of the 21 quantified metabolites and the 20 assigned lipid moieties is presented in Supplementary Table S6.

Analysis of serum macrophage-related cytokines, 21 quantified metabolites, and the assigned lipid moieties is presented in Supplementary Tables S7 and S8 for cranial GCA and PMR patients, respectively. Out of nine metabolites altered in the LV-GCA group, choline, and dimethyl sulfone were found to be significantly increased from the active to inactive disease states exclusively in the LV-GCA group (Figure 2A–C). The remaining metabolites displayed changes in the same directional trend across the disease states.

Next, we calculated ΔCholine values (the difference in choline levels between active and inactive disease) and ΔDimethyl sulfone values (the difference in dimethyl sulfone levels between active and inactive disease) in all three groups. The comparison of ΔCholine and ΔDimethyl sulfone among the three subgroups showed that only choline levels were significantly altered in the LV-GCA group. In contrast, the dimethyl sulfone difference between disease activity and inactivity was similar (Figure 2D).

### 3.3. Generation of Composite Indices for the Determination of Disease Activity/Remission

**Figure 3:**
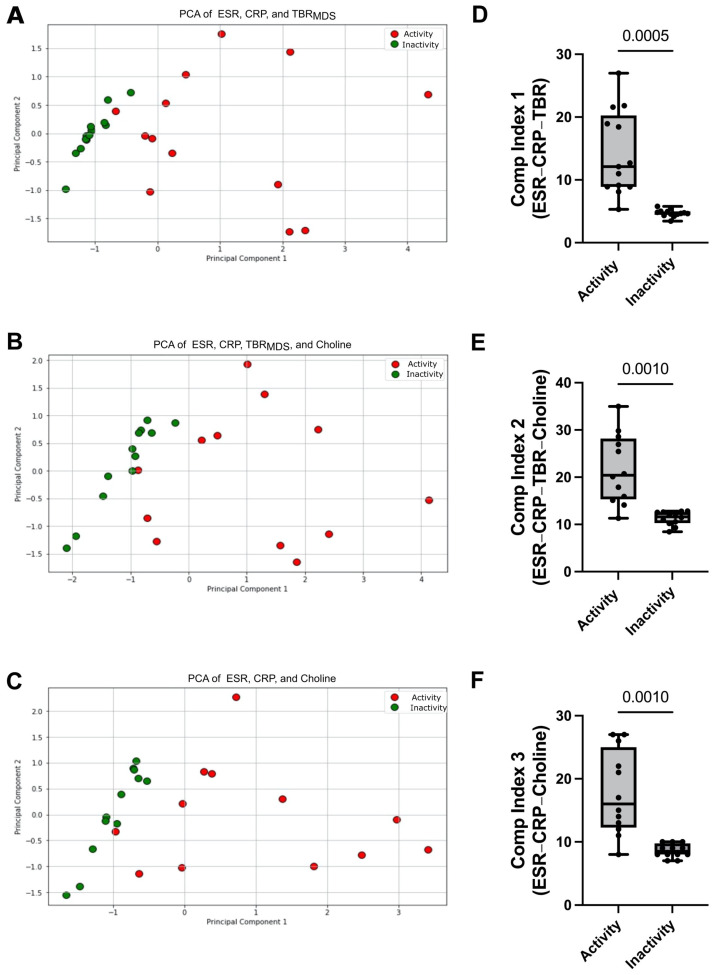
Principal Component Analysis (PCA) using the statistically significant parameters altered in large vessel vasculitis patients between active and inactive disease states shows discrimination between the two disease states irrespective of the combination of markers used. (**A**) PCA plots presenting the combination of ESR, CRP, and TBR_MDS_; (**B**) PCA plots presenting the combination of ESR, CRP, TBR_MDS,_ and choline; (**C**) PCA plots presenting the combination of ESR, CRP, and choline; (**D**) composite index 1 generation using ESR, CRP, and TBR_MDS_; (**E**) composite index 1 generation using ESR, CRP, TBR_MDS_, and choline; (**F**) composite index 3 generation using ESR, CRP, and choline. The first and third quartiles are shown in the lower and upper horizontal lines, respectively. The horizontal line in the boxes represents the median value. The exact *p* values are shown in each boxplot.

To develop a more accurate tool for disease activity, we performed PCA analysis using the various parameters found to be statistically significantly altered in the LV-GCA patients between activity and remission, such as CRP, ESR, TBR_MDS_, and choline levels. The first PCA model was a combination of TBR_MDS_ to CRP and ESR, resulting in a cumulative explained variance of 96% for the two principal components (Q^2^ = 0.94, 95CI^PC1^: 0.58 to 0.89, 95CI^PC2^: 0.09 to 0.37) (Figure 3A). The addition of choline to CRP, ESR, and TBR_MDS_ resulted in a cumulative explained variance of 84% for the two principal components (Q^2^ = 0.75, 95CI^PC1^: 0.50 to 0.77, 95CI^PC2^: 0.14 to 0.35) (Figure 3B). The addition of choline to CRP and ESR resulted in a cumulative explained variance of 96% for the two principal components (Q^2^ = 0.95, 95CI^PC1^: 0.55 to 0.83, 95CI^PC2^: 0.14 to 0.40) (Figure 3C). PCA analysis demonstrated that every combination of tested parameters can discriminate between the two phases of the disease.

Based on the findings of the PCA analysis, we sought to generate different composite indices to assess disease activity in LV-GCA patients. Composite index 1 (the sum of TBR_MDS_, CRP—categorical, and ESR—categorical) showed a statistically significant reduction from the active to inactive disease states (12.1 vs. 4.65, *p* = 0.0005) (Figure 3D). In addition, composite index 2 (the sum of TBR_MDS_, CRP—categorical, ESR—categorical, and choline—categorical) (20.4 vs. 11.62, *p* = 0.001) (Figure 3E) and composite index 3 (the sum of CRP—categorical, ESR—categorical, and choline—categorical) (16 vs. 8.5, *p* = 0.001) (Figure 3F) also showed a statistically significant decrease from the active to inactive disease. These data suggest that every combination of markers tested to generate a composite index of disease activity was able to separate the two groups effectively.

## 4. Discussion

In this proof-of-concept study, we have demonstrated the ability of 18F-FDG PET/CT imaging to distinguish disease activity and remission in LV-GCA patients and investigated the relationship between PET/CT findings with soluble mediators of inflammation using the TBR values of PET/CT. We found that metabolic inflammatory mediators’ levels (e.g., choline) rather than inflammatory cell-derived mediators’ (cytokines) levels are significantly altered while 18F-FDG-PET/CT-derived metrics are decreasing. Furthermore, choline levels were altered in the peripheral blood exclusively in the LV-GCA patient group and not in the inflammatory disease control groups consisting of PMR and cranial GCA patients with negative PET/CT scans. In addition, in LV-GCA patients, TBR and PETVAS were significantly decreased from the active to inactive phase. Finally, the composite index combining the traditional disease activity markers (ESR and CRP) and PET/CT-derived metrics along with choline levels was able to cluster patients more efficiently compared to only ESR-, CRP-, and PET/CT-based metrics.

Considering that in LV-GCA, the vascular wall is infiltrated by activated immune cells contributing to the signal intensity of PET/CT and that after glucocorticoid treatment there is a rapid switch of glycolysis to oxidative phosphorylation, it is conceivable that FDG can make a distinction between the active and inactive phase of the disease. However, in agreement with prior studies [12], we found a considerable overlap of TBR and PETVAS values between the two disease time points, since FDG uptake could also represent vascular remodeling and not immune cell activity only. To this end, more specific immuno-PET tracers are currently under investigation for vasculitis. By obtaining a deeper insight into how specific cell subpopulations are involved and behave in the pathogenesis of different forms of vasculitis, we may update our current tools for a more accurate assessment of disease activity [13,14].

In that direction, the activated fibroblast has attracted interest in the development of specific radiotracers [fibroblast activation protein inhibitor (FAPI PET/CT)] for the diagnosis and monitoring of LV-GCA [15]. Additionally, we found that altered choline levels are significantly increased moving from the active to inactive disease states, possibly due to an upregulated uptake of choline by tissue infiltrating macrophages. In-depth in vitro studies could provide further insights into the underlying mechanism and eventually support whether choline-based radiotracers could serve as alternative candidates to be utilized in the assessment of LV-GCA activity. Notably, in a murine model of atherosclerosis, 18F-choline could identify atherosclerotic plaques better compared to the traditional radiotracer 18FDG, a phenomenon attributed to the increased choline uptake by macrophages as shown by in vitro studies [16]. Contributing to the animal model findings, a study in stroke patients indicated an increase in 18F-fluorocholine (18F-FCH) uptake in the affected carotid plaques that correlated with CD68+ cells in the endarterectomy biopsies of the same patients [17].

Choline is an essential nutrient for cell structure and is involved in different biosynthesis pathways. Increased serum levels of choline have been associated with an increased cardiovascular disease risk [18], cerebrovascular disease risk [19], and increased risk of artery stenosis in hypertensive patients [20]. From a mechanistic perspective, macrophages, upon LPS (lipopolysaccharide) stimulation, demonstrate an increase in choline uptake and subsequent phosphatidylcholine synthesis, a biochemical process that renders them capable of proinflammatory cytokine production [21]. Choline can be transported into macrophages via the choline transporter-like protein-1 (CTL1). The inflammatory macrophage phenotype can be abrogated by CTL1 inhibition of choline uptake [22]. The above in vitro data suggest a central role of choline in macrophage metabolism and inflammatory signaling.

Previous studies have investigated the serum levels of several cytokines and chemokines in GCA and PMR patients [23]. IL-6 is considered the major cytokine in GCA pathogenesis that correlates with disease activity [24]; a finding that has not been validated in our study, among others, possibly due to the small sample size tested. The fact that serum levels of macrophage-related cytokines vary in different studies could be attributed to the different cohort sizes tested, but other reasons could also apply, including the different disease states and clinical phenotypes included in each study, the median time between treatment initiation and blood sampling, and the implementation of various analytical methods (ELISA, Luminex assay, and cytometric bead array) [25,26]. Consequently, different cytokine expression levels are reported in the literature [27]. Furthermore, their upregulated protein expression may be restricted to tissue injury. The restricted-to-tissue-injury production of cytokines has been shown recently in Takayasu arteritis tissue injury, where IL-6 protein expression was increased in the affected vessels and not in the peripheral blood of patients [28]. The above data suggest that the determination of accurate cytokine levels depends on a set of factors, and the results are not always representative of the disease state.

There is a limited number of studies investigating the serum metabolome in GCA and PMR patients [29]. Our previous work on NMR-based metabolomics in the serum of GCA and PMR patients yielded several metabolites and lipid moieties significantly altered from activity towards disease inactivity [11]. Among the results reported, N-acetyl glycoproteins (GlycA and GlycB) were identified as predictive markers of disease activity both in GCA and PMR, and choline was significantly altered in the GCA patient group, including both cranial GCA patients and LV-GCA patients. These results are also reported in the current analysis, which included a patient subset of our previous work. Moreover, choline levels were significantly altered only in the LV-GCA group, a finding that was not reported before since we did not subcategorize GCA patients based on the extent of the disease in the two groups of cranial and LV-GCA patients.

The strengths of this proof-of-concept study include encompassing different serum markers (cytokines, metabolites, lipids) along with normalized imaging data at two-time points of the disease in an attempt to extract novel next-generation biomarkers of disease activity [2]. Moreover, our study highlights the central role of immunometabolism in biomarker discovery, though further data enrichment is required. The limitations of our study include the small patient cohort used since we opted to include LV-GCA patients who have undertaken two PET/CT scans in a single PET/CT center to ensure consistency in the acquisition, processing, and interpretation of the images, which would have been challenging by different instrumentation.

## 5. Conclusions

In conclusion, the search for more sensitive disease activity biomarkers in LV-GCA can benefit from integrating patient clinical and imaging parameters with different omics technologies. Even if PET/CT is a sensitive method for assessing disease extent and activity, clinicians often face difficulties in everyday clinical practice. Thus, more studies, including wider patient cohorts, need to be conducted to extract novel biomarkers and composite indices capable of translating PET/CT imaging data into the fluid phase to increase the sensitivity of disease activity monitoring.

## Figures and Tables

**Table 1:** Baseline characteristics of all LV-GCA, cranial GCA, and PMR patients included in the study. In the parentheses, the interquartile range (IQR) is shown.

	LV-GCA (13)	Cranial-GCA (14)	PMR (14)
Age (median, years)	64 (60–75)	73 (65–81)	76 (63–79)
Gender (Female%, N)	69.2% (9/13)	64.3% (9/14)	57.1% (8/14)
Positive temporal artery biopsy	46.1% (6/13)	100% (14/14)	0% (0/14)
ESR active phase (median) (<20 mm/h)	55 (34.25–91.25)	80 (50–102)	68 (49–81)
ESR inactive phase (median) (<20 mm/h)	5 (3.75–12)	11 (9–15)	12.5 (6–19)
CRP active phase (median) (0–5 mg/L)	40 (8–119.37)	51 (13.8–91)	32 (20–49)
CRP inactive phase (median) (0–5 mg/L)	1 (0.45–1.73)	1.65 (0.6–4)	1.35 (1–3)
Hct active phase (median) (%)	35.5 (33.05–36.45)	37.2 (34–39.25)	38.4 (34.2–41.9)
Hct inactive phase (median) (%)	39.9 (37.45–41.8)	41.3 (39.98–44.18)	40 (36.4–44.4)
PLTs active phase (median)	355,000 (292,000–489,500)	387,000 (281,500–417,250)	337,000 (273,000–371,500)
PLTs inactive phase (median)	194,500 (173,000–238,000)	2,070,000 (194,750–324,500)	262,000 (210,000–290,000)
Arterial hypertension (%, N)	46% (6/13)	50% (7/14)	57.1% (8/14)
Diabetes mellitus (%, N)	15.4% (2/13)	35.7% (5/14)	28.5% (4/14)
Dyslipidemia (%, N)	61.5% (8/13)	42.8% (6/14)	71.4% (10/14)
BP-lowering drugs	38.4% (5/13)	50% (7/14)	50% (7/14)
Glucose-lowering drugs	15.4% (2/13)	35.7% (5/14)	21.4% (3/14)
Lipid-modifying drugs	38.4% (5/13)	42.8% (6/14)	57.1% (8/14)

ESR, Erythrocyte Sedimentation Rate; CRP, C-reactive protein; Hct, hematocrit; PLTs, platelets; BP, blood pressure.

## Data Availability

The original contributions presented in the study are included in the article/Supplementary Materials; further inquiries can be directed to the corresponding authors.

## Supplementary Materials

The following supporting information can be downloaded at: https://www.mdpi.com/article/10.3390/cells13221851/s1. Table S1: Assignment of lipid signals using the LED spectra. Peaks L2/L3, L5/L6 andL17/L18 are not resolved; Table S2: 21 Identified metabolites in the serum of GCA and PMR patients using the SMolESY platform; Table S3: Transformation of CRP, ESR and choline levels into categorical scale for composite index generation; Table S4: Comparison of metabolic activity assessed by 18-fluorodeoxyglucose (18F-FDG) positron emission tomography / computed tomography (PET/CT) and quantified by TBR on different aortic segments; Table S5: Macrophage-related cytokine levels of LVV patients in disease activity and inactivity along with *p*-values; Table S6: Disease activity markers, metabolites, and lipid moieties of LV-GCA patients in disease activity and inactivity along with *p*-values; Table S7: Disease activity markers, macrophage-related cytokines, metabolites, and lipid moieties of cranial-GCA patients in disease activity and inactivity along with *p*-values; Table S8: Disease activity markers, macrophage-related cytokines, metabolites, and lipid moieties of PMR in disease activity and inactivity along with *p*-values.
