# Supplementary material for: 18F-Fluorodeoxyglucose Positron Emission Tomography/Computed Tomography in Large-Vessel Vasculitis During Active and Inactive Disease Stages Is Associated with the Metabolic Profile, but Not the Macrophage-Related Cytokines: A Proof-of-Concept Study
Source: Cells. 2024 Nov 8;13(22):1851. doi: 10.3390/cells13221851 (PMC11592869; doi:10.3390/cells13221851)
Supplement: Supplementary file 1 [file cells-13-01851-s001.zip › cells-3269579-supplementary.pdf]

Supplementary Material

Article

18F-Fluorodeoxyglucose Positron Emission Tomography/Computed Tomography in Large-Vessel Vasculitis During Active and Inactive Disease Stages Is Associated with the Metabolic Profile, but Not the Macrophage-Related Cytokines: A Proof-of-Concept Study

Dimitris Anastasios Palamidas [1], Georgios Kalykakis [2,3], Dimitra Benaki [4], Loukas Chatzis [1,5,6], Ourania D. Argyropoulou [1], Panagiota Palla [1], Antonia Kollia [3], Pavlos Kafouris [3], Marinos Metaxas [3], Andreas V. Goules [1,5], Emmanuel Mikros [4,7], Konstantinos Kambas [8], Constantinos D. Anagnostopoulos [3,*] and Athanasios G. Tzioufas [1,5,6,*]

Citation: Palamidas, D.A.; Kalykakis, G.; Benaki, D.; Chatzis, L.; Argyropoulou, O.D.; Palla, P.; Kollia, A.; Kafouris, P.; Metaxas, M.; Goules, A.V.; et al. 18F-Fluorodeoxyglucose Positron Emission Tomography/Computed Tomography in Large-Vessel Giant Cell Arteritis During Active and Inactive Disease Stages Is Associated with the Metabolic Profile, but Not the Macrophage-Related Cytokines: A Proof-of-Concept Study. *Cells* **2024**, *13*, x. https://doi.org/10.3390/xxxxx

Academic Editor(s): Name

Received: 4 October 2024
Revised: date
Accepted: 5 November 2024
Published: date

Copyright: © 2024 by the authors. Submitted for possible open access publication under the terms and conditions of the Creative Commons Attribution (CC BY) license (https://creativecommons.org/licenses/by/4.0/).

1 Department of Pathophysiology, School of Medicine, National and Kapodistrian University of Athens, Athens, Greece.
2 Department of Informatics, Ionian University, Kerkyra, Greece.
3 PET-CT Department & Preclinical Imaging Unit, Center for Experimental Surgery, Clinical & Translational Research, Biomedical Research Foundation of the Academy of Athens, Athens, Greece.
4 Department of Pharmaceutical Chemistry, School of Pharmacy, National and Kapodistrian University of Athens, Athens, Greece.
5 Research Institute for Systemic Autoimmune Diseases, Athens, Greece.
6 Laboratory of Immunobiology, Center for Clinical, Experimental Surgery and Translational Research, Biomedical Research Foundation of the Academy of Athens, Athens, Greece.
7 Athena Research and Innovation Center in Information Communication & Knowledge Technologies, Marousi, 15125, Greece.
8 Laboratory of Molecular Genetics, Department of Immunology, Hellenic Pasteur Institute, Athens, Greece.

* Correspondence: cdanagnostopoulos@bioacademy.gr (CDA); agtzi@med.uoa.gr (AGT);

Supplementary Table S1:

Assignment of lipid signals using the LED spectra. Peaks L2/L3, L5/L6 andL17/L18 are not resolved.

Lipids	Signals	Assignment
L1	Lipids CH3- (cholesterol)	Cholesterol
L2	Lipids CH3- ; mainly HDL	Lipoproteins
L3	Lipids CH3-	Fatty acids
L4	Lipids CH3- (cholesterol)	Cholesterol
L5	Lipids CH2-	STOCSY with cholesterol
L6	Lipids CH2-	Fatty acids
L7	Lipids; FA C**H2**CH2C=C or C**H2**CH2C=O	Fatty acids
L8	FA -CH2-CH=	Fatty acids
L9	N-acetylglycoproteins (NCH3-)	GlycA
L10	N-acetylglycoproteins (NCH3-)	GlycB
L11	FA -CH2-C=O	Fatty acids
L12	FA C=CH-C**H2**-CH=C (overlapping)	
L13	N(CH3) choline head group	Choline group of bound phospholipids
L14	Glyceryl moieties of N-acetylglycoproteins	GlycA/GlycB
	Glyceryl moieties of N-acetylglycoproteins	GlycA/GlycB
L15	Glyceryl group CH2OCOR	TG, GPL (except LPC)
	Glyceryl group CH2OCOR	TG, GPL (except LPC)
L16	Glyceryl group CHOCOR	TG, GPL (except LPC)
L17	Cholesterol; Methine group CH=CH	Cholesterol
L18	Methine group CH=CH; Cholesterol	Unsaturated fatty acids

TG: triacylglycerols; GPL: Glycerophospholipids; LPC: lysophosphocholines

Supplementary Table S2:

21 Identified metabolites in the serum of GCA and PMR patients using the SMolESY platform.

**Metabolite**	**Metabolite**
3-Hydroxybutyric acid	Alanine
Acetic acid	Glutamine
Acetone	Histidine
Choline	Isoleucine
Citric acid	Leucine
Creatine	Lysine
Creatinine	Phenylalanine
Glucose	Tyrosine
Dimethyl sulfone	Valine
Glycine	Lactic acid
Formic acid	

Supplementary Table S3:

Transformation of CRP, ESR and choline levels into categorical scale for composite index generation. CRP and ESR levels are increased during active disease resulting to an increased categorical value. Choline levels are decreased during active disease compared to inactive disease resulting to reversed categorical values.

**CRP SCALE**	**CRP CATEGORY**	**ESR SCALE**	**ESR CATEGORY**	**CHOLINE SCALE**	**CHOLINE CATEGORY**
<= 5	1	≤ 20	1	≤ 6000	9
>5 to ≤ 20	2	> 20 to ≤ 35	2	> 6000 to≤ 9000	8
> 20 to ≤ 40	3	> 35 to ≤ 50	3	> 9000 to ≤ 12000	7
> 40 to ≤ 50	4	> 50 to ≤ 60	4	> 12000 to ≤ 15000	6
> 50 to ≤ 60	5	> 60 to ≤ 70	5	> 15000 to ≤ 18000	5
> 60 to ≤ 70	6	> 70 to ≤ 80	6	> 18000 to ≤ 21000	4
> 70 to ≤ 80	7	> 80 to ≤ 90	7	> 21000 to ≤ 24000	3
> 80 to ≤ 90	8	> 90 to ≤ 100	8	> 24000 to ≤ 28000	2
> 90	9	> 100	9	> 28000	1

Supplementary Table S4:

Comparison of metabolic activity assessed by 18-fluorodeoxyglucose (18F-FDG) positron emission tomography / computed tomography (PET/CT) and quantified by TBR on different aortic segments. In the parenthesis, the interquartile range (IQR) is shown.

Pair	n	Activity	Inactivity	
		Median	Median	P value
TBR_{MDS}	13	3.321 (2.946 – 4.9)	2.655 (2.49 – 2.985)	**0.0061**
TBR_{GLOBAL}	13	2.712 (2.360 – 3.118)	2.442 (2.025 – 2.627)	**0.0479**
TBR_{ABD}	13	2.852 (2.487 – 4.031)	2.406 (2.101 – 2.721)	**0.0171**
TBR_{ARC}	13	2.576 (2.21 – 3.477)	2.499 (2.013 – 2.6)	**0.0398**
TBR_{ASC}	13	2.579 (2.34 – 3.415)	2.351 (2.058 – 2.754)	0.0574
TBR_{DSC}	13	2.64 (2.33 – 3.489)	2.356 (1.984 – 2.651)	**0.0215**

TBR_{MDS}, target-to-background ratio of the most diseased aortic segment; ABD, suprarenal and infrarenal abdominal aorta; ARC, aortic arch; ASC, ascending aorta; DSC, descending aorta;

Supplementary Table S5

Macrophage-related cytokine levels of **LVV patients** in disease activity and inactivity along with p-values. In the parenthesis the interquartile range (IQR) is shown.

Pair	n	Activity	Inactivity	
		Median	Median	P value
**Macrophage-related cytokines (pg/ml)**				
**IFN-γ**	10	25.44 (0 - 324.26)	25.44 (0 - 121.7)	0.4375
**IL-10**	10	1.62 (1.08 - 2.24)	1.62 (0.8 - 1.62)	0.4609
**IL-12p40**	10	0.96 (0.48 - 8.55)	0.96 (0.48 - 2.45)	0.4688
**IL-12p70**	10	1.02 (0.41 - 3.87)	0.49 (0.49 - 3.59)	1
**IL-1RA**	10	1.16 (0.49 - 9.07)	1.16 (0.49 - 3.02)	0.375
**IL-1β**	10	0.55 (0.41 - 18.66)	0.55 (0.41 - 18.66)	0.1875
**IL-23**	10	1.11 (1.11 - 42.39)	2.6 (1.11 - 8.7)	0.375
**TARC**	10	91.11 (44.39 - 211)	76.83 (56.36 - 138.17)	0.3125
**TNF-α**	10	0.48 (0.36 - 12.4)	3.22 (0.36 - 4.33)	0.4375
**IL-4**	10	1.44 (0.51 - 7.53)	0.92 (0.51 - 3.23)	0.2969
**IL-6**	10	5.21 (1.84 - 10.24)	16.2 (5.21 - 19.67)	0.3828
**IP-10**	10	103.62 (79.48 - 130.55)	82.79 (63.86 - 120.52)	0.3828
**Arginase**	10	4864.61 (2882.15 - 6009.84)	3158.21 (1969.82 - 5472.08)	0.4609

Supplementary Table S6

Disease activity markers, metabolites, and lipid moieties of **LV-GCA patients** in disease activity and inactivity along with p-values. In the parenthesis the interquartile range (IQR) is shown. AU, Arbitrary Units;

Pair	n	Activity	Inactivity	
		Median	Median	P value
**Disease activity markers**				
**TBR_{MDS}**	13	3.321 (2.946 - 4.9)	2.655 (2.49 - 2.985)	**0.0061**
**CRP (mg/L)**	13	40 (8 - 119.37)	1 (0.45 - 1.73)	**0.0002**
**ESR (mm/h)**	13	55 (34.25 - 91.25)	5 (3.75 - 12)	**0.0005**
**Metabolites (AU)**				
**3_Hydroxybutyric_acid**	12	149264.077 (96017.083 – 277574.251)	75337.221 (19403.553 – 159936.638)	0.0771
**Acetic acid**	12	30675.115 (22404.831 - 44968.147)	36452.373 (24500.227 - 41668.619)	1
**Acetone**	12	44889.751 (22141.412 - 73686.166)	39531.153 (22648.689 - 51874.437)	0.4961
**Alanine**	12	279401.629 (232985.671 - 330836.485)	436317.922 (348155.683 - 514088.231)	**0.0034**
**Choline_Smoothed**	12	9437.318 (8025.092 - 12060.932)	11331.028 (9178.770 - 13287.796)	**0.021**
**Citric acid**	12	87586.041 (12112.084 - 129531.201)	71943.929 (0 - 133398.610)	0.8457
**Creatine**	12	14016.959 (8744.659 - 30480.198)	26989.571 (13588.854 - 46130.914)	0.2661
**Creatinine_Smoothed**	12	42179.336 (39355.233 - 53154.316)	53502.868 (48630.188 - 59155.689)	0.064
**Dimethyl sulfone**	12	11459.921 (7853.698 - 14308.611)	17281.863 (11431.339 - 31199.270)	**0.0342**
**Formic_acid**	12	30486.133 (8478.613 - 48691.678)	23606.894 (0 - 60578.784)	0.8501
**Glucose**	12	5363184.683 (4648658.890 - 5653730.606)	5558267.191 (4661157.037 - 7340105.782)	0.1099
**Glutamine**	12	644778.108 (407773.624 - 744415.070)	635327.164 (530839.482 - 780657.323)	0.2334
**Glycine**	12	185838.727 (154624.985 - 244871.712)	220604.84 (168820.210 - 278366.847)	0.1294
**Histidine_Smoothed**	12	57556.375 (45302.764 - 61856.401)	48123.702 (42868.155 - 64772.634)	0.7334
**Lactic_acid**	12	1360712.832 (1115823.754 - 1943843.782)	1676772.245 (1185332.791 - 2434780.937)	0.3804
**Leucine**	12	130112.585 (103560.240 - 146698.931)	121100.397 (90395.623 - 142484.957)	0.9097
**Lysine**	12	31783.503 (14001.725 - 100100.470)	75292.627 (56903.107 - 145957.312)	0.2334
**Phenylalanine_Smoothed**	12	70282.2 (63582.078 - 81444.113)	71549.375 (59694.089 - 75883.685)	0.9097
**Tyrosine_Smoothed**	12	53635.202 (45807.116 - 65751.600)	60706.92 (52760.268 - 78107.405)	0.2036

**Valine**	12	229507.207 (192114.815 - 262554.039)	255642.197 (231424.244 - 278646.999)	0.1294
**Isoleucine**	12	94926.154 (87248.903 - 101366.681)	93610.005 (78058.774 - 110409.922)	0.9097
**Lipids (AU)**				
**L1** Cholesterol	12	0.0119 (0.0116 - 0.0122)	0.012 (0.0113 - 0.0123)	0.7334
**L2** Lipoproteins (mainly HDL)	12	0.0497 (0.0476 - 0.0544)	0.0543 (0.0499 - 0.0586)	**0.0342**
**L3** Fatty acids	12	0.0308 (0.0290 - 0.0327)	0.0324 (0.0298 - 0.0391)	0.0923
**L4** Cholesterol	12	0.0406 (0.0403 - 0.0410)	0.0395 (0.0377 - 0.0408)	0.0522
**L5** Fatty acids	12	0.0556 (0.0548 - 0.0595)	0.0637 (0.0599 - 0.0663)	**0.0093**
**L6** Fatty acids	12	0.127 (0.115 - 0.135)	0.143 (0.127 - 0.187)	0.0522
**L7** Fatty acids	12	0.0242 (0.023 - 0.0244)	0.025 (0.0235 - 0.0276)	0.0522
**L8** Fatty acids	12	0.04393 (0.0430 - 0.0452)	0.04502 (0.0447 - 0.0463)	0.0977
**L9** GlycA	12	0.0102 (0.00893 - 0.0112)	0.00742 (0.00703 - 0.00787)	**0.0161**
**L10** GlycB	12	0. 00811 (0.00728 - 0.0089)	0. 00586 (0.00544 - 0.00611)	**0.0093**
**L11** Fatty acids	12	0.0229 (0.0221 - 0.0235)	0.0242 (0.0222 - 0.0263)	0.064
**L12** Fatty acids	12	0.0281 (0.0272 - 0.0291)	0.0271 (00.0263 - 0.0279)	0.1294
**L13** Choline group of bound phospholipids	12	0.0223 (0.0212 - 0.0241)	0.0255 (0.0236 - 0.0276)	0.0977
**L14A** GlycA/GlycB	12	0.0322 (0.0275 - 0.036)	0.0205 (0.0192 - 0.0228)	0.0547
**L14B** GlycA/GlycB	12	0.0561 (0.0522 - 0.0660)	0.0411 (0.0374 - 0.0444)	0.0742
**L15A** TG, GPL (except LPC)	12	0.0076 (0.00736 - 0.00772)	0.00764 (0.00756 - 0.00779)	0.8501
**L15B** TG, GPL (except LPC)	12	0.00728 (0.00710 - 0.00741)	0.00737 (0.00724 - 0.00748)	0.7344
**L16** TG, GPL (except LPC)	12	0.00147 (0.0014 - 0.00162)	0.00161 (00.00137 - 0.0022)	0.0923
**L17** Cholesterol	12	0.0159 (0.0152 - 0.0173)	0.0178 (0.0170 - 0.0198)	**0.0093**
**L18** Unsaturated fatty acids	12	0.0156 (0.0134 - 0.0159)	0.0181 (0.0151 - 0.0203)	**0.0342**

Supplementary Table S7

Disease activity markers, macrophage-related cytokines, metabolites, and lipid moieties of **cranial-GCA patients** in disease activity and inactivity along with p-values. In the parenthesis the interquartile range is shown. AU, Arbitrary Units;

Pair	n	Activity	Inactivity	
		Median	Median	P value
**Disease activity markers**				
**CRP (mg/L)**	14	51 (13.8 - 91)	1.65 (0.6 - 4)	**0.0001**
**ESR (mm/h)**	14	80 (50 - 102)	11 (9 - 15)	**0.0004**
**Macrophage-related cytokines (pg/ml)**				
**IFN-γ**	10	0 (0 - 52.24)	0 (0 - 0)	0.125
**IL-10**	10	1.62 (1.09 - 2.66)	1.18 (0.8 - 1.62)	**0.0469**
**IL-12p40**	10	0.55 (0.26 - 0.96)	0.26 (0.26 - 0.26)	**0.0313**
**IL-12p70**	10	0.49 (0.49 - 1.02)	0.49 (0.32 - 0.49)	0.125
**IL-1RA**	10	0.59 (0.39 - 1.16)	0.59 (0.39 - 1.16)	0.2324
**IL-1β**	10	0.55 (0.55 - 4.01)	0.55 (0 - 0.55)	0.0569
**IL-23**	10	1.11 (0.25 - 2.6)	0.68 (0.25 - 1.11)	**0.0469**
**IL-4**	10	0.51 (0.51 - 1.44)	0.51 (0.51 - 1.44)	0.6875
**IL-6**	10	5.21 (2.95 - 12.07)	2.03 (0.97 - 5.87)	0.2783
**IP-10**	10	123.11 (90.23 - 140.63)	93.21 (41.19 - 123.84)	0.1763
**TARC**	10	350.77 (88.72 - 848.13)	293.66 (89.91 - 358.5)	0.0522
**TNF-α**	10	0.48 (0 - 0.48)	0.48 (0 - 0.48)	0.5625
**Arginase**	10	5408.21 (4327.6 - 6075.99)	5176.04 (1434.63 - 8357.34)	0.5186
**Metabolites (AU)**				
**3_Hydroxybutyric acid**	14	105136.15 (87927.863 - 129261.657)	56433.462 (19146.203 - 117838.264)	0.1189
**Acetic acid**	14	22278.466 (15060.478 - 33380.38)	34430.126 (12736.313 - 42766.212)	0.8077
**Acetone**	14	41962.691 (20257.925 - 57384.509)	33314.508 (26836.399 - 43396.528)	0.4263
**Alanine**	14	336159.99 (294821.306 - 383450.006)	402671.98 (357693.61 - 507566.675)	**0.004**
**Choline_Smoothed**	14	9977.45 (7551.121 - 12929.498)	10957.704 (9455.691 - 15177.196)	0.2958
**Citric acid**	14	80736.718 (54039.036 - 125539.686)	101402.62 (9161.787 - 140472.694)	0.9032
**Creatine**	14	27255.897 (17017.115 - 34297.943)	36066.213 (14744.137 - 39422.902)	0.9032
**Creatinine_Smoothed**	14	43163.915 (40249.513 - 57087.410)	50867.718 (44104.796 - 61598.776)	0.2958

**Dimethyl_sulfone**	14	13627.986 (10097.959 - 20378.841)	18393.915 (12552.789 - 20917.196)	0.3258
**Formic_acid**	14	19035.779 (8423.181 - 54142.940)	23033.921 (8860.151 - 36413.18)	0.8552
**Glucose**	14	5341851.6 (4380296.083 - 9256952.378)	4658111.8 (4240193.605 - 5813084.128)	0.1189
**Glutamine**	14	592213.84 (337651.536 - 690590.473)	627816.82 (472311.9 - 683488.282)	0.5016
**Glycine**	14	214923.53 (181810.460 - 242575.296)	183216.28 (140539.239 - 207628.111)	0.1726
**Histidine_Smoothed**	14	44624.05 (31526.663 - 60114.866)	50495.22 (43846.809 - 61064.546)	0.3258
**Lactic acid**	14	1882331 (1691931.610 - 2148586.466)	2431963.9 (1568097.690 - 2787817.694)	0.2166
**Leucine**	14	127936.48 (104799.900 - 143955.514)	135008.51 (116418.111 - 139247.851)	0.1937
**Lysine**	14	89272.128 (24838.951 - 179122.504)	83231.408 (43158.192 - 180756.836)	0.583
**Phenylalanine_Smoothed**	14	83572.339 (74319.541 - 94667.303)	73060.595 (63796.171 - 81707.560)	0.0785
**Isoleucine**	14	109586.36 (101863.870 - 122273.377)	91171.957 (51384.503 - 118734.627)	0.4631
**Tyrosine_Smoothed**	14	57597.207 (50272.753 - 64580.278)	66143.304 (37726.753 - 75432.951)	0.5416
**Valine**	14	267315.51 (230226.534 - 294161.467)	250289.18 (215301.342 - 304195.785)	0.6257
**Lipids (AU)**				
**L1** Cholesterol	14	0.0115 (0.0111 - 0.0118)	0.0115 (0.0109 - 0.0117)	0.3258
**L2** Lipoproteins (mainly HDL)	14	0.0496 (0.0457 - 0.0530)	0.0518 (0.0499 - 0.0544)	**0.0001**
**L3** Fatty acids	14	0.032 (0.0297 - 0.0349)	0.0352 (0.0340 - 0.0388)	**0.004**
**L4** Cholesterol	14	0.0399 (0.0388 - 0.0410)	0.0381 (0.0368 - 0.0396)	**0.0245**
**L5** Fatty acids	14	0.0572 (0.0549 - 0.0577)	0.0623 (0.0598 - 0.0636)	**0.0001**
**L6** Fatty acids	14	0.135 (0.128 - 0.145)	0.157 (0.152 - 0.184)	**0.0017**
**L7** Fatty acids	14	0.0243 (0.0232 - 0.0251)	0.0264 (0.0258 - 0.0273)	**0.0009**
**L8** Fatty acids	14	0.0444 (0.0431 - 0.045)	0.0463 (0.0455 - 0.0469)	**0.0017**
**L9** GlycA	14	0.00975 (.00934 - 0.0111)	0.00778 (0.00749 - 0.00801)	**0.0001**
**L10** GlycB	14	0.00772 (0.00734 - 0.00888)	0.00594 (0.00555 - 0.00621)	**0.0001**
**L11** Fatty acids	14	0.0232 (0.0230 - 0.0243)	0.0253 (0.0247 - 0.0262)	**0.0085**
**L12** Fatty acids	14	0.0282 (0.0264 - 0.0301)	0.0273 (0.0258 - 0.0280)	0.0785
**L13** Choline group of bound phospholipids	14	0.0219 (0.0192 - 0.0231)	0.0246 (0.0240 - 0.0257)	**0.0001**
**L14A** GlycA/GlycB	14	0.0311 (0.029 - 0.0365)	0.0211 (0.0204 - 0.0223)	**0.0001**
**L14B** GlycA/GlycB	14	0.0292 (0.0270 - 0.0355)	0.0211 (0.0204 - 0.0224)	**0.002**

**L15A** TG, GPL (except LPC)	14	0.00759 (0.0074 - 0.00784)	0.00768 (0.0076 - 0.00791)	0.3258
**L15B** TG, GPL (except LPC)	14	0.00746 (0.00717 - 0.00750)	0.00741 (0.00724 - 0.00758)	0.7695
**L16** TG, GPL (except LPC)	14	0.0017 (0.0016 - 0.00174)	0.00191 (0.00184 - 0.0022)	**0.0067**
**L17** Cholesterol	14	0.016 (0.0152 - 0.0179)	0.018 (0.0169 - 0.0188)	**0.0085**
**L18** Unsaturated fatty acids	14	0.0158 (0.0150 - 0.0176)	0.0195 (0.018 - 0.0205)	**0.0067**

Supplementary Table S8

Disease activity markers, macrophage-related cytokines, metabolites, and lipid moieties of **PMR** in disease activity and inactivity along with p-values. In the parenthesis, the interquartile range (IQR) is shown; AU, Arbitrary Units;

Pair	n	Activity	Inactivity	
		Median	Median	P value
**Disease Activity Markers**				
**CRP (mg/L)**	14	32 (20-49)	1.35 (1- 3)	**0.0002**
**ESR (mm/h)**	14	68 (49 - 81)	12.5 (6 - 19)	**0.0001**
**Macrophage-related cytokines (pg/ml)**				
**IFN-γ**	7	25.44 (0 - 220.84)	25.44 (6.36 - 120.43)	0.8125
**IL-10**	7	0.8 (0.8 - 1.51)	0.8 (0.57 - 2.28)	0.8125
**IL-12p40**	7	0.96 (0.26 - 13.25)	0.55 (0.333 - 2.63)	1
**IL-12p70**	7	0.49 (0.235 - 3.13)	0.49 (0.24 - 3.13)	0.25
**IL-1RA**	7	2.6 (0.59 - 4.94)	1.16 (0.29 - 3.03)	0.4375
**IL-1β**	7	0.55 (0.55 - 16.01)	0.55 (0.55 - 3.15)	0.3125
**IL-23**	7	4.64 (0.250 - 11.217)	1.11 (0.465 - 6.54)	0.875
**IL-4**	7	1.44 (0.742 - 9.535)	1.44 (0.742 - 9.11)	0.625
**IL-6**	7	2.95 (0.95 - 6.19)	4.01 (1.512 - 6.19)	0.9375
**IP-10**	7	103.62 (63.1 - 228.09)	107.62 (82.44 - 387.25)	0.6875
**TARC**	7	165.92 (101.73 - 432.61)	242.55 (83.53 - 374.98)	0.8125
**TNF-α**	7	0.48 (0 - 20.09)	0.48 (0.12 - 2.54)	1
**Arginase**	7	4169.34 (2929.18 - 6660.47)	4980.4 (4199.01 - 8201.53)	**0.0469**
**Metabolites (AU)**				
**3 Hydroxybutyric acid**	14	124462.97 (94855.453 - 170950.580)	131270.98 (103986.602 - 183768.312)	0.6698
**Acetic acid**	14	30155.6 (16113.624 - 67694.531)	20569.889 (18814.436 - 28426.194)	0.104
**Acetone**	14	42368.93 (28755.070 - 48029.887)	38141.444 (33912.491 - 49385.573)	0.7148
**Alanine**	14	389540.12 (308083.603 - 430919.581)	387406.38 (305384.039 - 463212.343)	0.9032
**Choline_Smoothed**	14	9361.858 (7791.787 - 16651.798)	10464.009 (7852.177 - 13955.958)	1
**Citric acid**	14	34011.999 (20774.935 - 78143.687)	125329.09 (0-141777.118)	0.0785
**Creatine**	14	25689.035 (20187.971 - 33288.744)	25292.156 (23229.314 - 53491.564)	0.2958

**Creatinine_Smoothed**	14	43398.873 (34210.994 - 48250.584)	49509.203 (41930.548 - 55858.355)	**0.0494**
**Dimethyl sulfone**	14	11076.771 (10085.603 - 13093.144)	15750.66 (12584.044 - 17905.011)	0.3575
**Formic acid**	14	35695.376 (21238.552 - 53178.257)	24200.925 (14541.871 - 33744.188)	**0.0134**
**Glucose**	14	6413606.4 (4738573.904 - 7474745.032)	5526216.2 (5006654.523 - 7353283.135)	0.6257
**Glutamine**	14	609093.77 (497901.853 - 750492.496)	713328.82 (516963.337 - 856948.730)	0.6257
**Glycine**	14	207927.27 (119599.651 - 238415.088)	175209.2 (156767.209 - 225457.957)	0.9032
**Histidine_Smoothed**	14	48242.739 (38465.482 - 58747.897)	52275.844 (46310.185 - 62185.737)	0.4631
**Lactic_acid**	14	1571956.8 (1406682.957 - 2633280.248)	1600354.3 (1474800.538 - 2496008.892)	0.8552
**Leucine**	14	126110.51 (97900.301 - 145769.353)	131240.61 (109402.274 - 146005.076)	0.6257
**Lysine**	14	133295.73 (54540.395 - 187664.548)	128448.62.83 (73135.056 - 185495.381)	0.7609
**Phenylalanine_Smoothed**	14	76399.268 (71527.247 - 95151.093)	66364.772 (60399.077 - 79857.214)	**0.0419**
**Tyrosine_Smoothed**	14	57707.845 (50714.558 - 61587.722)	63060.221 (57474.738 - 66947.502)	0.5416
**Valine**	14	265134.17 (247293.819 - 309909.008)	246340.75 (237063.280 - 297979.507)	0.3575
**Isoleucine**	14	107423.29 (90407.221 - 115170.825)	104444.7 (88416.350 - 119274.022)	0.7148
**Lipids (AU)**				
**L1** Cholesterol	14	0.0119 (0.0117 - 0.0124)	0.0121 (0.0113 - 0.0126)	0.2958
**L2** Lipoproteins (mainly HDL)	14	0.0495 (0.0458 - 0.0514)	0.0546 (0.0519 - 0.0568)	**0.0006**
**L3** Fatty acids	14	0.0313 (0.0299 - 0.0332)	0.0315 (0.0309 - 0.0328)	0.4631
**L4** Cholesterol	14	0.0412 (0.0393 - 0.0422)	0.0405 (0.0388 - 0.0414)	0.7148
**L5** Fatty acids	14	0.0547 (0.0515 - 0.0576)	0.0619 (0.0589 - 0.0656)	**0.0001**
**L6** Fatty acids	14	0.127 (0.110 - 0.158)	0.135 (0.116 - 0.146)	0.6698
**L7** Fatty acids	14	0.0245 (0.0231 - 0.0259)	0.0247 (0.0236 - 0.0259)	0.391
**L8** Fatty acids	14	0.0429 (0.0428 - 0.0449)	0.0446 (0.0441 - 0.0450)	**0.0419**
**L9** GlycA	14	0.00967 (0.00887 - 0.0118)	0.00788 (0.00774 - 0.00826)	**0.0001**
**L10** GlycB	14	0.00761 (0.00660 - 0.00869)	0.00646 (0.00641 - 0.00659)	**0.0004**
**L11** Fatty acids	14	0.0236 (0.0214 - 0.0251)	0.0237 (0.0222 - 0.0252)	0.7148
**L12** Fatty acids	14	0.0279 (0.0267 - 0.0284)	0.028 (0.0264 - 0.0289)	0.4263
**L13** Choline group of bound phospholipids	14	0.0225 (0.0191 - 0.0233)	0.0249 (0.0224 - 0.0261)	**0.0006**
**L14A** GlycA/GlycB	14	0.0284 (0.0246 - 0.0368)	0.0235 (0.0227 - 0.0242)	**0.0012**
**L14B** GlycA/GlycB	14	0.0551 (0.0471 - 0.0640)	0.0452 (0.0429 - 0.0484)	**0.0469**
**L15A** TG, GPL (except LPC)	14	0.00782 (0.00758 - 0.00842)	0.00755 (0.00724 - 0.00817)	0.0906
**L15B** TG, GPL (except LPC)	14	0.00748 (0.00736 - 0.00778)	0.00709 (0.00705 - 0.00747)	0.0781
**L16** TG, GPL (except LPC)	14	0.00148 (0.00126 - 0.00201)	0.00162 (0.00130 - 0.00184)	0.8077
**L17** Cholesterol	14	0.0152 (0.013 - 0.0165)	0.0179 (0.0168 - 0.0190)	**0.0001**

**L18** Unsaturated fatty acids	14	0.0155 (0.0128 - 0.0167)	0.0161 (0.0150 - 0.0181)	0.2412
